# Supplementary material for: Dynamics of influenza transmission in vampire bats revealed by longitudinal monitoring and a large-scale anthropogenic perturbation
Source: Sci Adv. 2025 Feb 5;11(6):eads1267. doi: 10.1126/sciadv.ads1267 (PMC11797540; doi:10.1126/sciadv.ads1267)
Supplement: Supplementary file 1 — Figs. S1 to S9 Tables S1 to S3 [file sciadv.ads1267_sm.pdf]

Science Advances
AAAS

Supplementary Materials for

Dynamics of influenza transmission in vampire bats revealed by longitudinal monitoring and a large-scale anthropogenic perturbation

Megan E. Griffiths *et al.*

Corresponding author: Megan E. Griffiths, megan.Griffiths@glasgow.ac.uk, m3griffiths@gmail.com

Sci. Adv. **11**, eads1267 (2025)
DOI: 10.1126/sciadv.ads1267

This PDF file includes:

Figs. S1 to S9
Tables S1 to S3

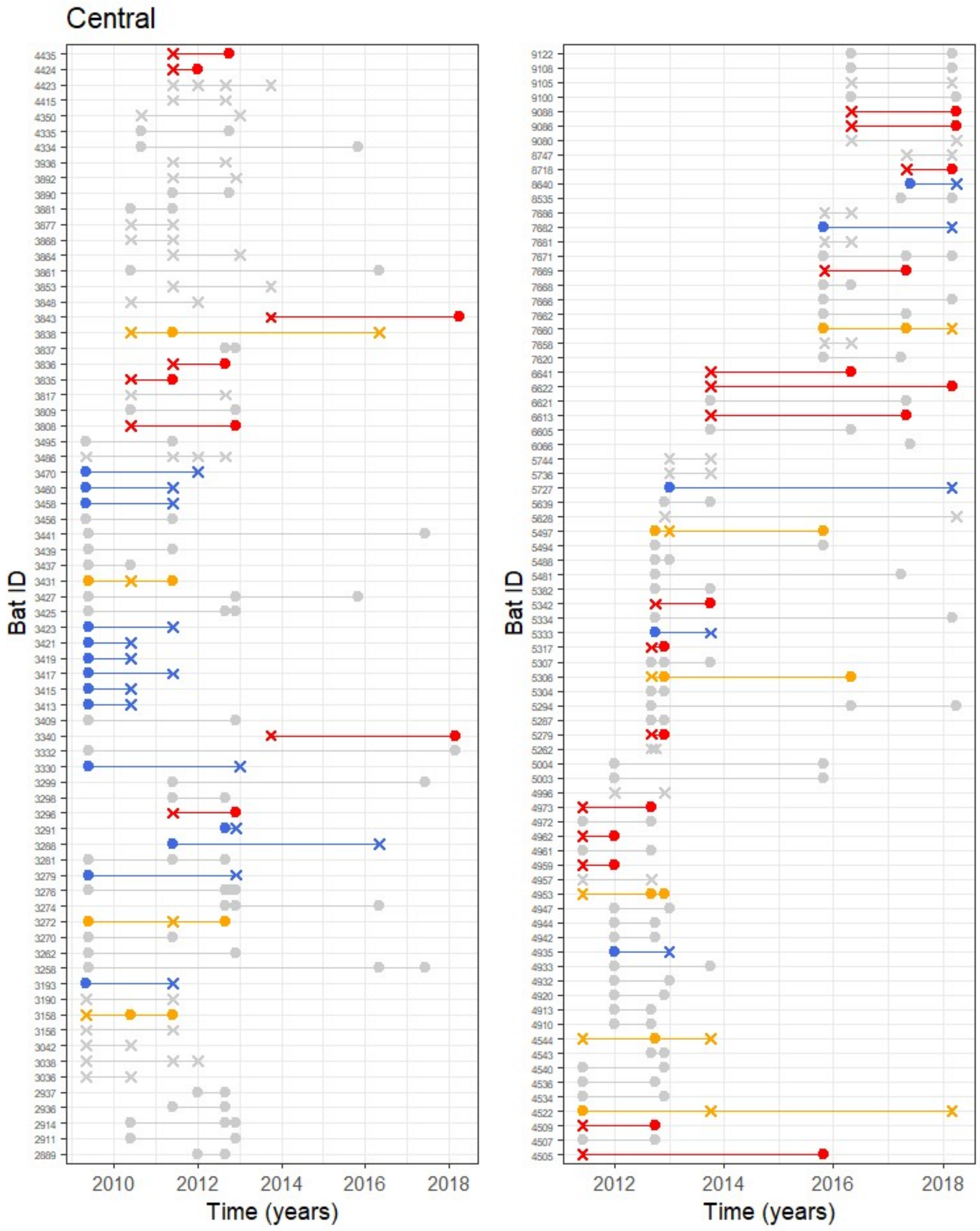
Central
Bat ID
Time (years)
2010
2012
2014
2016
2018
Bat ID
Time (years)
2012
2014
2016
2018

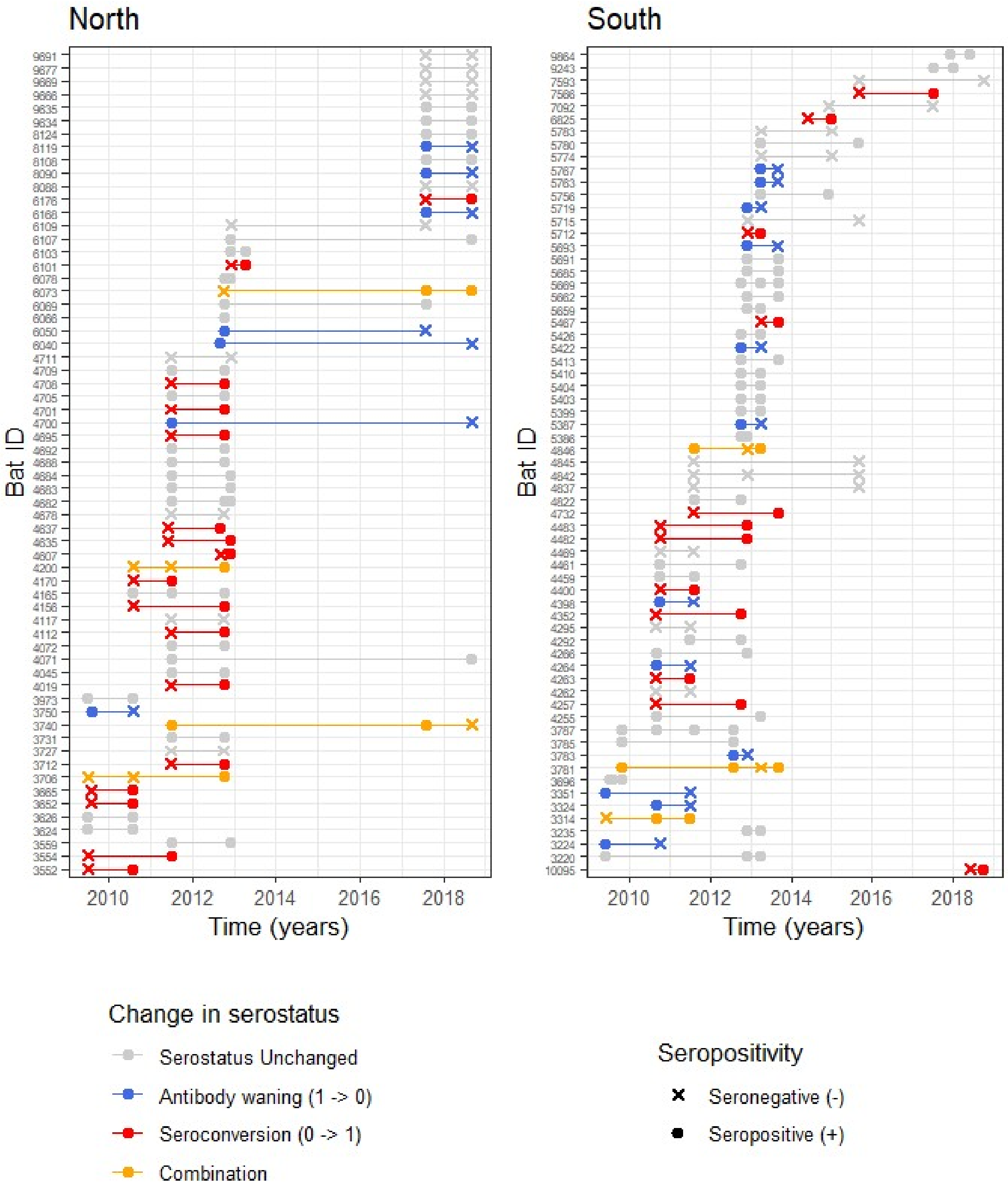

Fig. S1. Longitudinal serology data from recaptured bats for each zone (Central N=149; North N=63; South N=65).

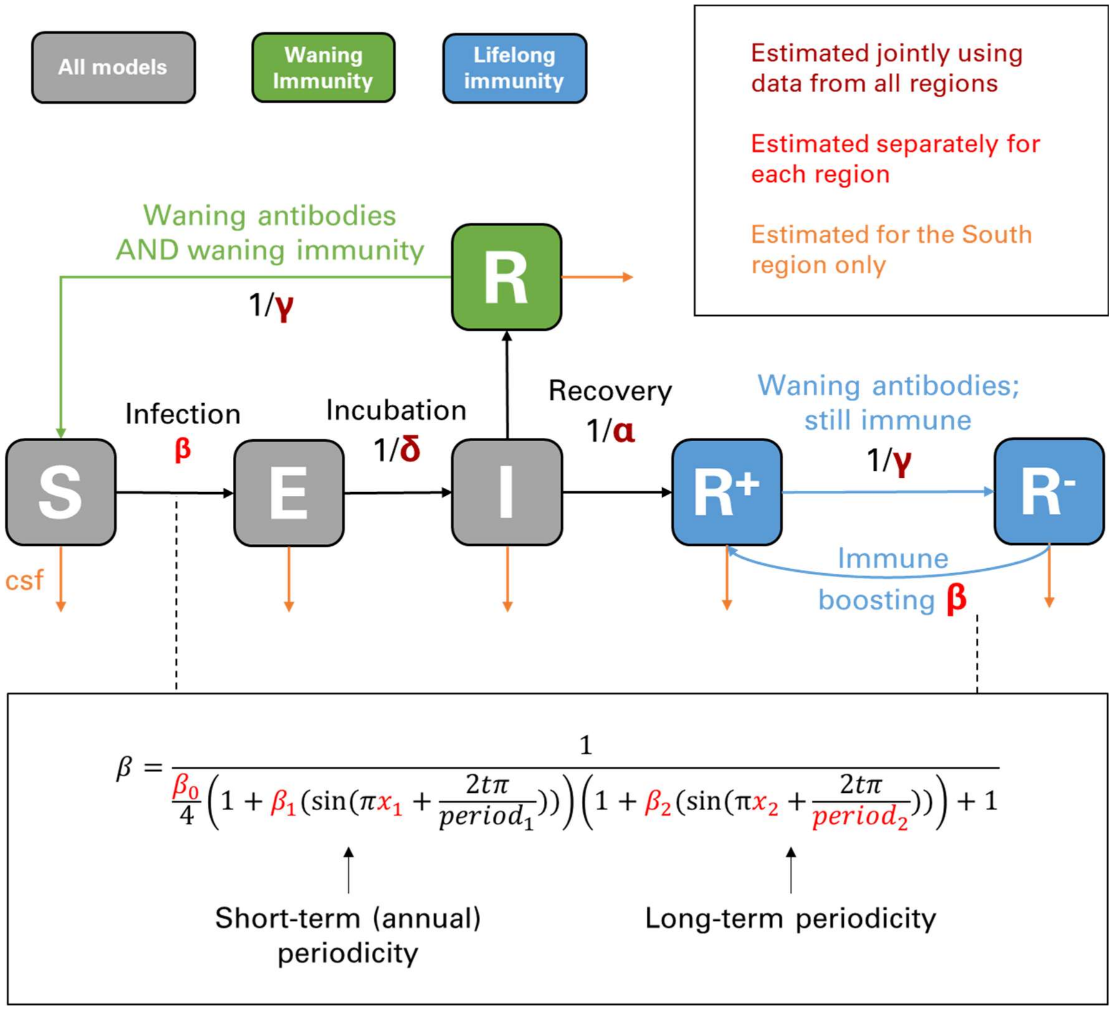

$$\beta = \frac{1}{\frac{\beta_0}{4}\left(1 + \beta_1(\sin(\pi x_1 + \frac{2t\pi}{period_1}))\right)\left(1 + \beta_2(\sin(\pi x_2 + \frac{2t\pi}{period_2}))\right) + 1}$$

Fig. S2. Schematic of BIV compartmental models for both waning and lifelong immunity. Grey compartments and black arrows show the states/rates of transitions that are common to all models/zones (S=Susceptible, E=Exposed, I=Infected). Green shows the states and transitions for the model assuming waning immunity where R=Recovered and seropositive. Blue shows the states and transitions for the model assuming lifelong immunity where R^+=Recovered and seropositive and R^-=Recovered but seronegative.

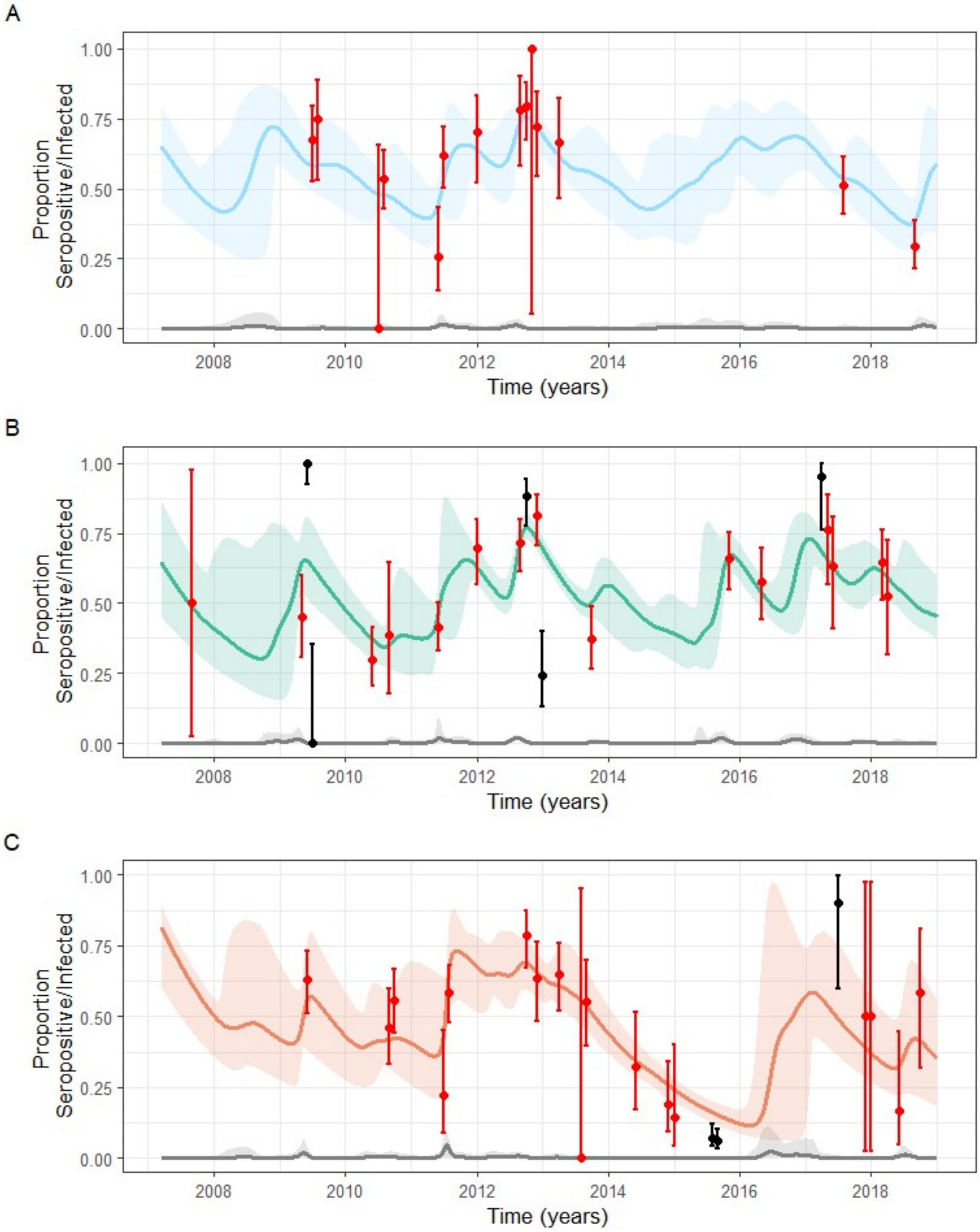

Fig. S3. Bat Influenza virus seroprevalence data and posterior predictive distributions when assuming lifelong rather than waning immunity. Population-level seroprevalence data with binomial confidence intervals per month for populations in (A) North, (B) Central, and (C) South are shown with colours indicating whether the observed seropositivity falls inside (red) or outside (black) the 95% CI of the posterior cumulative distribution function. The mean trajectory for the Recovereds fit by the model is shown for each population by the coloured solid line, and the 95% CIs by the shaded regions. The mean trajectory for the Infecteds fit by the model is shown for each population by the grey line and shaded region.

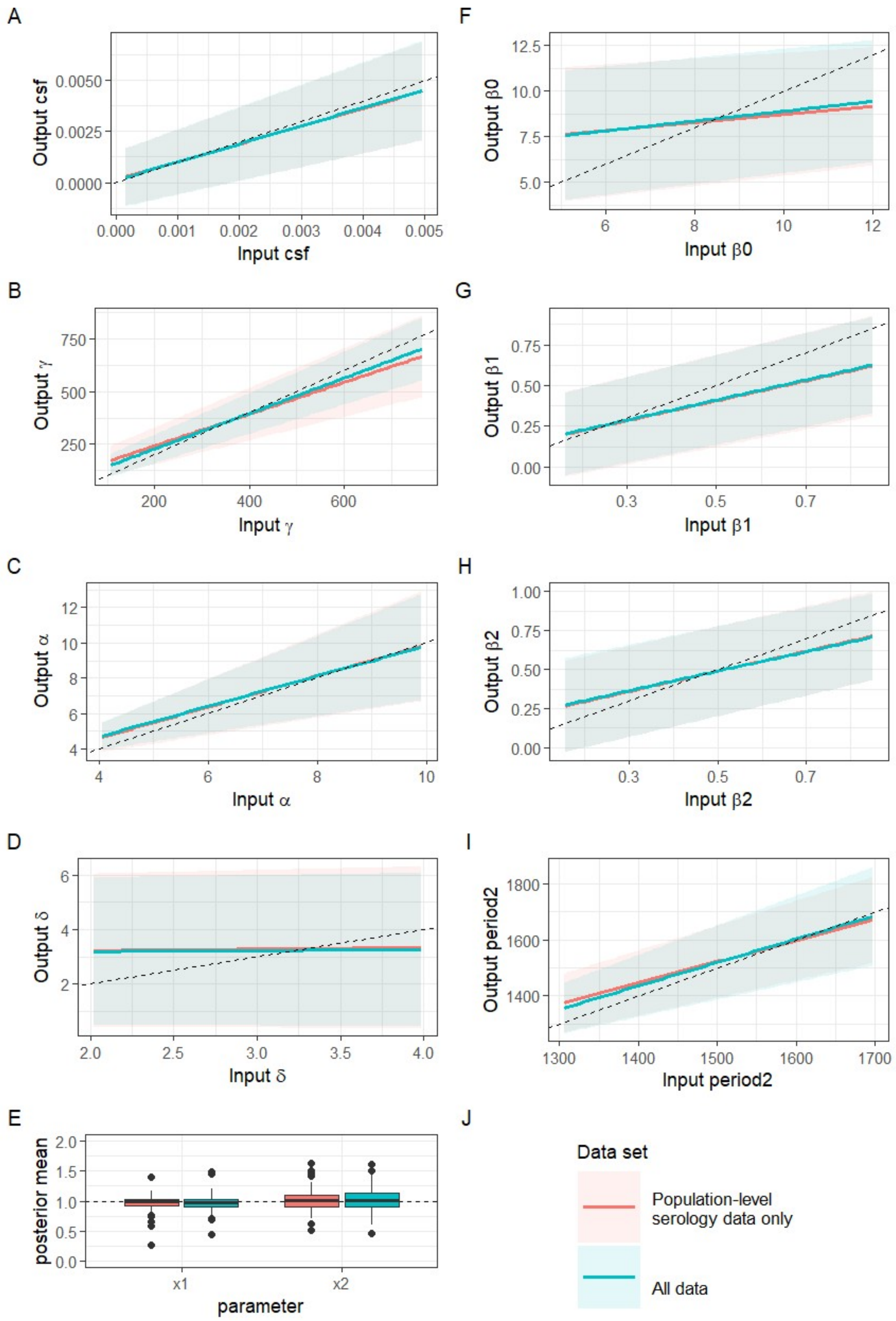

Fig. S4. Model fitting to test data and data inclusion comparison. The x-axis of each graph shows the input parameter value, and the y-axis shows the mean of the posterior distribution for that parameter when model fitting to test data. The shaded areas show 95% credible intervals. The dotted line on each plot is at x=y where the model prediction matches the input value of the estimated parameter. Mean lines which are closer to this dotted line show better prediction by the model.

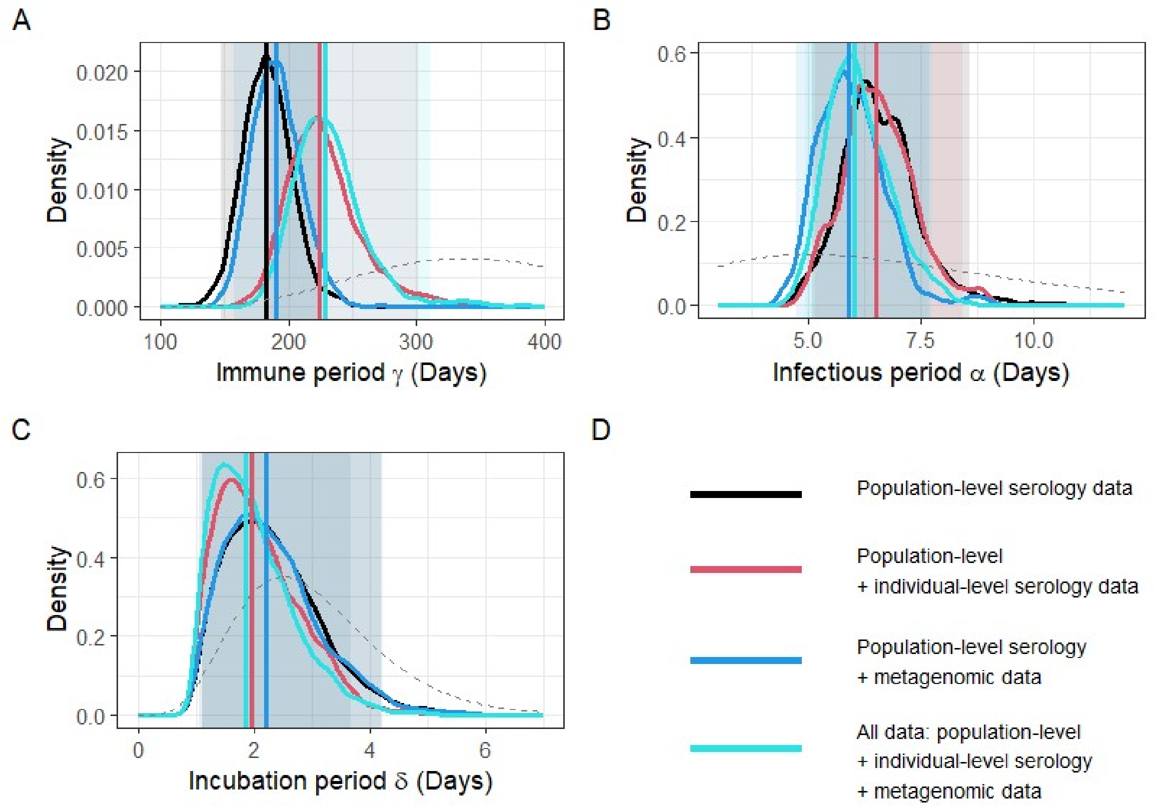

Fig. S5. Comparison of the posterior distributions of jointly estimated parameters when different data types are included in the model. Plots show the posterior mean and distribution when using different combinations of data, including population-level serology, individual-level seroconversion histories, and metagenomic data. Vertical lines show the mean of the distribution, and shaded areas show the 95% credible intervals. The dashed lines show the prior distribution for each parameter.

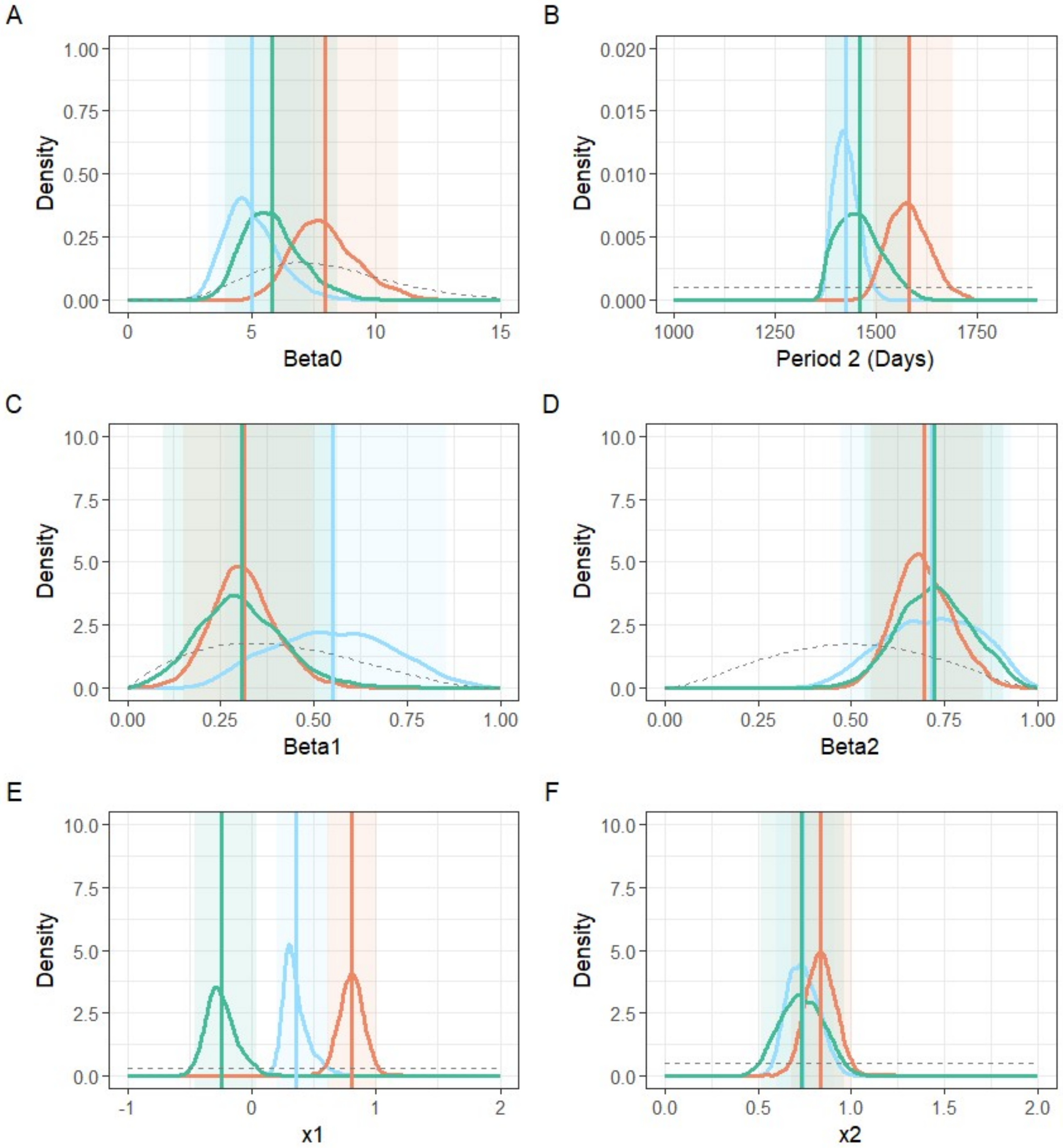

Fig. S6. Posterior distributions for all parameters within the formula for the rate of transmission β (Equation 11). β = base rate of transmission, beta1 = magnitude of short-term cycle peaks, beta2 = magnitude of long-term cycle peaks, period2 = the time between peaks of the long-term cycles, x1 = timing of the short-term cycles, and x2 = timing of the long-term cycles. Posterior means for each zone (blue=North; green=Central; orange=South) are shown by the vertical lines, and 95% credible intervals are shown by the shaded areas. The dashed lines show the prior distribution for each parameter.

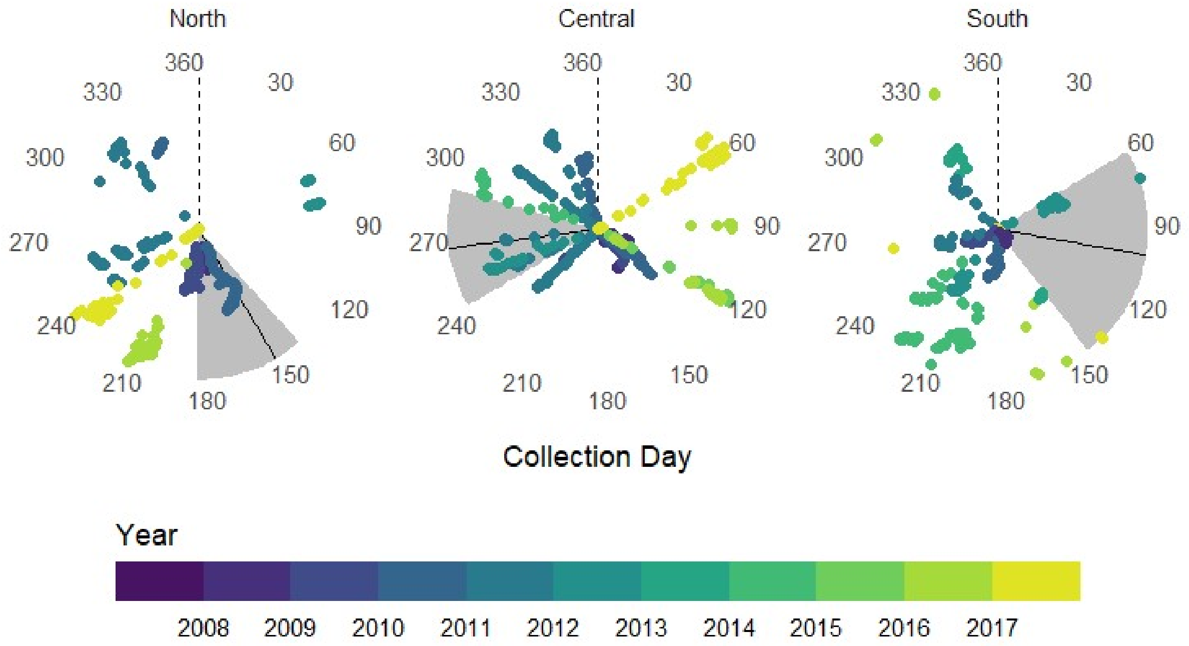

Fig. S7. Timing of sample collection throughout the year across the 12-year study period, in each zone. Each point represents an individual bat sample. The shaded areas show the upper and lower quartiles of the estimated peak in infected bats for each region, showing that these estimates are not simply correlated with peaks in sample collection.

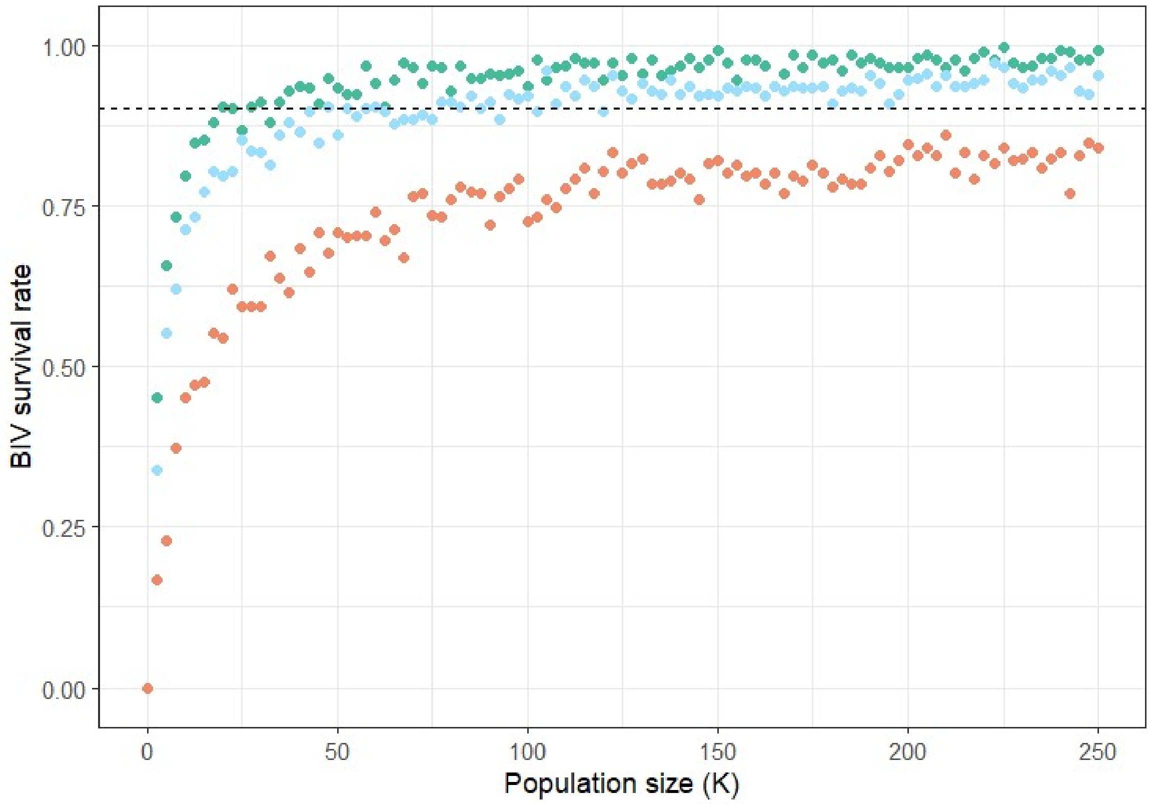

Fig. S8. Population size required for long term maintenance of BIV in vampire bat populations. Points show the proportion of stochastic simulations (out of 250 simulations) in which BIV survives for the full sampling period in each zone (green=Central; blue=North; orange=South). The dashed line shows the population size needed for 95% of simulations to result in BIV survival, where at least one Infected or Exposed bat is present in the population at the end of the 12-year simulation. The larger population size requirement in the South reflects the impact of culling.

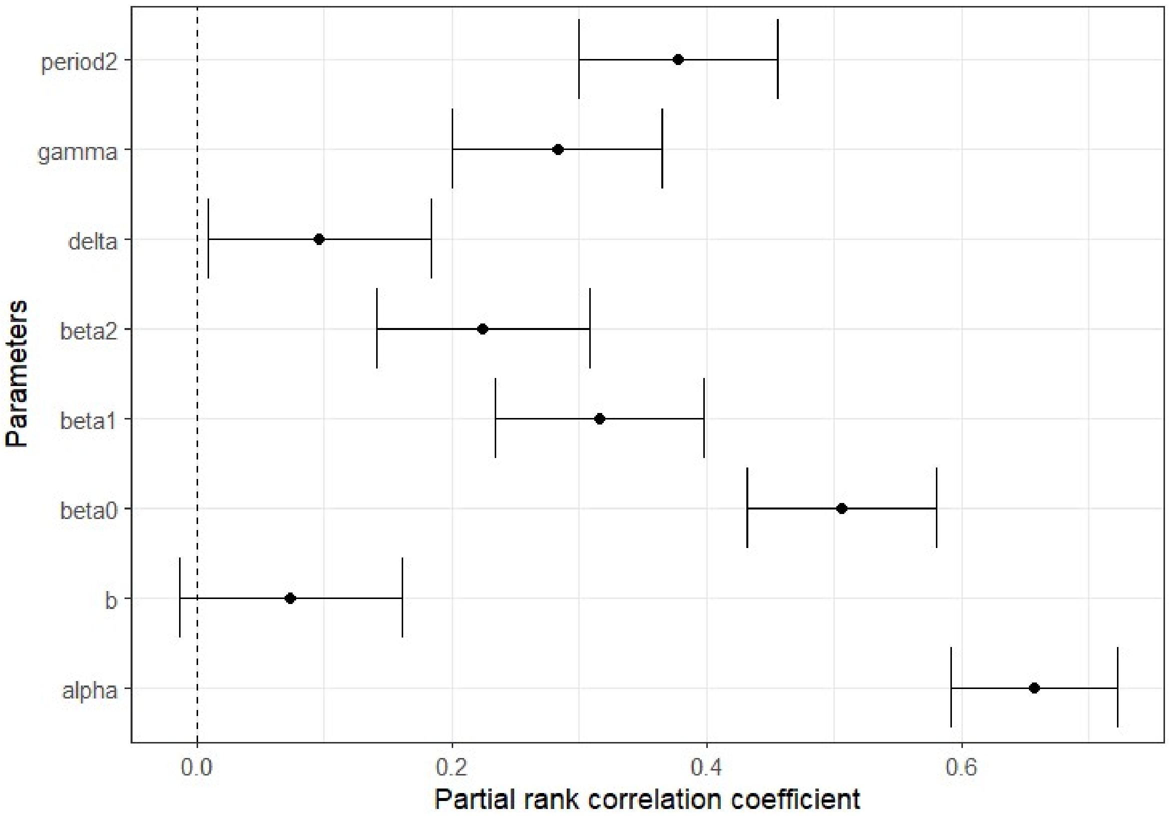

Fig. S9. Partial rank correlation coefficients (PRCC) for key model parameters. Dots show the mean PRCC estimates averaged across each timepoint of the model output, and lines show the bounds of the 95% CIs.

Table S1. Details of all sites from which bats samples used in this study were taken. DR = *Desmodus rotundus*. Other bat species includes sites where species have been observed to co-roost; A & S specify that *Sturnira* and *Artibeus sp*. were observed at a site. Where Direct culling refers to sample sites within areas where Vampiricide was applied to captured bats.

Site abbreviation	Region	Zone	Years sampled	Total DR sampled	Total DR positive	Sero-prevalence	Other bat species	Direct culling
AMA1	Amazonas	North	2011	13	10	0.77	Y – A & S	N
AMA2	Amazonas	North	2011-2013	36	21	0.58	Y	N
AMA3	Amazonas	North	2011	3	2	0.67	N	N
API1	Apurimac	South	2009-2015, 2017-2018	227	119	0.52	N	Y
API13	Apurimac	South	2009-2015	115	71	0.62	Y - S	Y
API140	Apurimac	South	2010-2015, 2017	161	59	0.37	N	N
API141	Apurimac	South	2015	45	5	0.11	N	Y
API15	Apurimac	South	2015	7	0	0	N	N
API16	Apurimac	South	2015	14	0	0	N	Y
API17	Apurimac	South	2015	31	1	0.03	N	Y
API18	Apurimac	South	2015	9	2	0.22	N	Y
API3	Apurimac	South	2010-2013	70	39	0.56	N	Y
API9	Apurimac	South	2009-2013	80	46	0.58	N	Y
AYA1	Ayacucho	South	2015	15	0	0	N	Y
AYA11	Ayacucho	South	2015	23	5	0.22	Y - A	N
AYA13	Ayacucho	South	2015	6	1	0.17	N	Y
AYA14	Ayacucho	South	2015	32	4	0.13	N	Y
AYA15	Ayacucho	South	2015	29	1	0.03	N	Y
AYA4	Ayacucho	South	2015	53	1	0.02	N	Y
AYA7	Ayacucho	South	2015	6	0	0	N	Y
CAJ1	Cajamarca	North	2009-2012, 2017-2018	117	64	0.55	Y	N
CAJ2	Cajamarca	North	2009-2012, 2017-2018	181	115	0.64	Y	N
CAJ3	Cajamarca	North	2009-2012, 2017-2018	118	59	0.50	Y	N
CAJ4	Cajamarca	North	2010-2013, 2017-2018	146	72	0.49	Y	N
CUS6	Cusco	South	2015	31	0	0	Y	N
CUS8	Cusco	South	2015	25	1	0.04	N	N
LMA10	Lima	Central	2007, 2009-2013, 2015	130	30	0.23	N	N
LMA4	Lima	Central	2009-2013, 2015, 2017-2018	171	125	0.73	Y - A	N
LMA5	Lima	Central	2009-2013, 2015-2018	221	118	0.53	N	N
LMA6	Lima	Central	2009-2013, 2015-2018	436	296	0.68	N	N

Table S2. Posterior parameter estimates (mean and SD) for different levels of data inclusion. Cells coloured green show that the change in the mean estimate for a parameter from that estimated by the base data set is greater than the SD for that estimate, with yellow cells showing a change greater than SD/2. Parameters estimated separately for each zone are shown in the order: North, South, Central.

Parameter	Population-level serology data		Population + individual-level serology data		Population-level serology + metagenomic data		All data	
	Mean	SD	Mean	SD	Mean	SD	Mean	SD
δ	2.33	0.81	2.18	0.72	2.32	0.83	1.99	0.70
α	6.58	0.86	6.55	0.79	5.95	0.75	6.12	0.72
γ	183.44	19.49	227.89	29.27	190.85	19.06	232.43	30.21
beta0	5.98	1.43	5.50	1.18	5.39	1.28	5.00	1.07
	9.12	1.67	8.70	1.42	7.92	1.41	7.97	1.32
	6.55	1.30	6.51	1.40	5.71	1.17	5.83	1.17
beta1	0.56	0.16	0.53	0.15	0.52	0.17	0.55	0.16
	0.36	0.09	0.31	0.09	0.36	0.09	0.31	0.09
	0.27	0.11	0.30	0.12	0.28	0.11	0.31	0.12
beta2	0.74	0.12	0.73	0.12	0.75	0.12	0.72	0.13
	0.68	0.08	0.68	0.08	0.70	0.08	0.69	0.08
	0.70	0.10	0.70	0.10	0.73	0.10	0.72	0.10
period 2	1413.53	27.55	1420.29	29.39	1414.10	30.21	1424.35	28.69
	1591.15	49.04	1581.94	48.42	1590.31	58.61	1579.80	50.15
	1430.79	60.35	1450.57	65.87	1431.54	60.03	1459.16	54.12
x1	0.38	0.12	0.37	0.11	0.36	0.14	0.35	0.11
	0.77	0.10	0.80	0.10	0.77	0.10	0.81	0.11
	-0.26	0.14	-0.22	0.15	-0.28	0.13	-0.24	0.13
x2	0.72	0.08	0.73	0.08	0.72	0.09	0.74	0.08
	0.86	0.08	0.84	0.08	0.85	0.10	0.84	0.09
	0.69	0.13	0.71	0.15	0.69	0.13	0.74	0.12
csf	0.0024	8.12E-4	0.0025	8.10E-4	0.0024	7.71E-4	0.0023	8.30E-4

Table S3. Comparison and evaluation of the models assuming waning and lifelong immunity.

	**Waning Immunity**		**Lifelong immunity**
Structure	SEIR	SEIR – no short-term periodicity	$SEIR^+R^-$
DIC	836.90	878.12	923.22
looic estimate	867.30	926.10	1013.40
looic se	85.60	88.00	101.40
elpd-loo estimate	-433.70	-463.00	-506.70
elpd-loo se	42.80	44.00	50.70
Max Rhat	1.02	1.03	2.76
